# Intracranial Inoculation Is More Potent Than Intranasal Inoculation for Inducing Optic Neuritis in the Mouse Hepatitis Virus-Induced Model of Multiple Sclerosis

**DOI:** 10.3389/fcimb.2018.00311

**Published:** 2018-09-04

**Authors:** Manmeet Singh, Reas S. Khan, Kimberly Dine, Jayasri Das Sarma, Kenneth S. Shindler

**Affiliations:** ^1^Department of Biological Science, Indian Institute of Science Education and Research Kolkata, Mohanpur, India; ^2^FM Kirby Center for Molecular Ophthalmology, Scheie Eye Institute, University of Pennsylvania, Philadelphia, PA, United States

**Keywords:** optic neuritis, demyelination, mouse hepatitis virus, intranasal inoculation, intracranial inoculation

## Abstract

Neurotropic strains of mouse hepatitis virus (MHV) induce acute inflammation and chronic demyelination in the spinal cord and optic nerves mediated by axonal spread following intracranial inoculation in mice, with pathologic features similar to the human demyelinating disease multiple sclerosis. Spinal cord demyelination is also induced following intranasal inoculation with neurotropic MHV strains, however much higher viral doses are required as compared to intracranial inoculation. Recently, it was shown that intranasal administration of low concentrations of proteins leads to significant, rapid accumulation of protein in the optic nerve and in the eye, with only low levels reaching spinal cord and other brain regions. Thus, we examined whether intranasal inoculation with MHV at doses equivalent to those given intracranially could induce optic neuritis—inflammation, demyelination and loss of retinal ganglion cells (RGCs) in the optic nerve with or without inducing spinal cord demyelination. Four week old male C57BL/6J mice were inoculated intracranially with the recombinant demyelinating strain RSA59, or intranasally with RSA59 or the non-demyelinating strain RSMHV2 as control. One month post-inoculation, mice inoculated intracranially with RSA59 had significant myelin loss in both spinal cord and optic nerves, with significant loss of RGCs as well, consistent with prior studies. As expected, intranasal inoculation with RSA59 failed to induce demyelination in spinal cord; however, it also did not induce optic nerve demyelination. No acute inflammation was found, and no viral antigen was detected, in the optic nerve or retina 1 day after inoculation. Results confirm the neurotropic effects of RSA59 following intracranial inoculation, and suggest that direct infection with axonal transport of virus from brain to spinal cord and optic nerve is required to induce demyelinating disease. These studies suggest that MHV does not selectively concentrate in optic nerve and retina to sufficient levels to induce demyelination following intranasal inoculation. Intracranial inoculation should continue to be considered a preferred method for studies of MHV-induced optic neuritis and central nervous system (CNS) demyelinating disease.

## Introduction

Neurotropic strains of MHV have been extensively used to induce neuroinflammation mediated acute and chronic demyelinating disease of CNS. Depending upon route of inoculation and strain of MHV, different regions of the CNS are affected. Inoculation with experimental neurotropic strains JHM and MHV-A59 induces a biphasic disease with acute meningoencephalitis (in first 10–14 days post inoculation) followed by subacute and chronic inflammatory demyelinating disease (Stohlman and Weiner, 1981; Lavi et al., 1984; Das Sarma et al., 2000). Both JHM and MHV-A59 strains of MHV cause some subacute and chronic inflammatory demyelination in the brain, but a much larger disease burden in the spinal cord. Translocation of virus from initial site of inoculation in brain to spinal cord occurs by trafficking of virus particle in neural and glial cells (Perlman et al., 1995; Sun and Perlman, 1995).

Intranasal as well as intracranial inoculation of JHM has been shown to induce similar symptoms in BALB/C mice (Robbins et al., 1991). Similarly, intracranial inoculation has been well used as a method for MHV-A59 to cause the biphasic disease (Das Sarma et al., 2000, 2002), while a higher dose of MHV-A59 is required to reach the same level of inflammation in CEACAM-/- mice when inoculated through the nasal route (Blau et al., 2001; Hemmila et al., 2004). With intracranial inoculation, the inflammation is not limited to brain and spinal cord. MHV-A59 and recombinant strain RSA59 cause inflammation in optic nerve with subsequent demyelination of optic nerve and RGC loss (Shindler et al., 2008, 2011). Studies of isogenic recombinant strains RSA59 and RSMHV2 of demyelinating strain (MHV-A59) and non-demyelinating strain (MHV2), respectively, containing enhanced green fluorescent protein (EGFP) have elaborated the mechanisms of demyelination and axonal loss and have helped in tracking and tracing of virus *in vitro* as well as *in vivo* (Das Sarma et al., 2009). RSA59 can cause demyelination, but RSMHV2 cannot, which makes it a suitable control to determine the cellular and molecular basis of demyelination in mice.

Following intranasal inoculation of mice, MHV accesses the CNS through the olfactory nerve and spreads from the olfactory system (Jacobsen and Perlman, 1990; Perlman et al., 1990) into structures of the limbic system and their brainstem connections. This has led investigators to suggest that interneuronal transport is one mechanism of viral spread during acute encephalitis (Barthold, 1988; Lavi et al., 1988; Barnett et al., 1993), and studies showing spread of virus sequentially from cerebral hemispheres to brainstem to spinal cord (Perlman et al., 1990) provide further support for this interneuronal transport mechanism. Similar axonal transport of virus from brain to spinal cord (Das Sarma et al., 2009), as well as from brain to optic nerve (Shindler et al., 2008, 2011), has been reported following intracranial inoculation with MHV-A59 or RSA59 and may serve as one mechanism for virus to avoid immune surveillance; however, axonal spread to optic nerve has not been well examined following intranasal inoculation.

Different intra- and extracellular pathways may help facilitate viral transport across olfactory or respiratory epithelial barriers. Endocytosis into olfactory sensory neurons followed by intraneuronal transport to the olfactory bulb, or transcellular transport to the lamina propria via sustentacular cells, have been suggested as potential intracellular pathways (Kristensson and Olsson, 1971; Broadwell and Balin, 1985; Thorne et al., 1995; Doty, 2008; Kristensson, 2011). Delivery of large molecular weight biological therapies (e.g., stem cells, gene therapy vectors, and large proteins) to the CNS via intranasal administration has been explored as a potential method to treat multiple CNS diseases/disorders including Parkinson's and Alzheimer's diseases, multiple sclerosis, seizures, strokes, and psychiatric disorders (Costantino et al., 2007; Neuwelt et al., 2008; Dhuria et al., 2010). Spread of smaller peptides through rodent brain following intranasal administration occurs rapidly, with diffuse brain distribution and greatest levels found in olfactory bulbs and trigeminal nerves, just 1 h after treatment. IGF-1 (*MW* = 7.65 kDa) is one of the most studied proteins using intranasal delivery to the CNS (Thorne et al., 2004). Even entry of some high molecular weight proteins such as VEGF (MW = 38.2 kDa) to the CNS has been shown following intranasal administration (Yang et al., 2009). Recently, it has been shown that proteins in a complex biologic therapy, ST266, administered via the intranasal route in rats reached the CNS within 30 min, and ST266 proteins were detected in the vitreous and the optic nerve at markedly higher concentrations than in the brain (Khan et al., 2017), suggesting a rapid, direct nose-to-optic nerve delivery method for proteins. Whether viruses can follow similar pathways to preferentially spread to optic nerve at low inoculation titers has not been reported, but if such pathways are present, the rapid spread of virus could provide an additional mechanism for immune evasion and therefore promote viral infection at lower inoculation titers.

We hypothesized that RSA59 can be used to induce optic neuritis when inoculated intranasally at lower doses than required to induce brain and spinal cord disease due to enhanced viral spread to optic nerve. Mice were inoculated with RSA59 and RSMHV2 as control, both intranasally as well as intracranially at equivalent concentrations to compare if both routes of administration result in the same optic nerve pathology.

## Materials and methods

### Mice

Four-week-old virus-free C57BL/6J mice were purchased from the Jackson Laboratory (BarHarbor, ME, USA). All animal procedures and care were conducted in accordance with ethical guidelines approved by the Institutional Animal Care and Use Committee at the University of Pennsylvania.

### Viruses

RSA59 and RSMHV2, the isogenic recombinant strain of MHV-A59 and MHV2, respectively, were used as previously described (Das Sarma et al., 2009). RSA59 and RSMHV2 are each engineered to express enhanced green fluorescence protein (EGFP), thus allowing viral antigen detection by fluorescence without immunohistochemical staining (Das Sarma et al., 2002). Mice were monitored daily for signs of MHV induced neurologic up to 26 days (chronic stage) post-infection.

### Inoculation of mice

50% LD50 doses of strains RSA59 (70,000 PFU), and RSMHV2 (1000 PFU) were used to inoculate 4-week-old, MHV-free, C57BL/6J mice (Jackson Laboratory). Desired PFU of viruses were prepared in a total volume of 20 μl in PBS and were pipeted as intranasal drops noninvasively every 2 min to alternating nares until all 20 μl were delivered, with simultaneous occlusion of the opposite naris. Drops were placed at the opening of the nares, allowing them to be snorted into the nasal cavity. The mice for day 26 studies were also inoculated intracranially as a positive control for disease pathogenesis with RSA59, as in prior studies (Das Sarma et al., 2009). Control mice mock-infected with PBS were inoculated in parallel. Animals were euthanized (3 mice per group) at day 1 post-inoculation (p.i.) and day 26 p.i.

### Histopathology

At 1 and 26 days p.i., tissues, including whole eyes, optic nerves, brains, spinal cords, and livers, were isolated from both mock- and virus-infected mice. For paraffin sectioning, eyes and optic nerves were fixed for 15 min after dissecton in 4% paraformaldehyde (PFA) while brains, spinal cords, and livers were fixed in 4% PFA overnight. Five micrometer sections were cut for routine CNS pathology staining following fixation and tissue processing. Sections were stained with Luxol Fast Blue (LFB) to detect myelin loss in spinal cord and optic nerve as in prior studies (Shindler et al., 2008). Demyelination was scored based on detection of focal white matter areas lacking LFB staining using a relative three-point scale. Areas of demyelination were quantified using a 0–3 point scale, where 0-no demyelination; 1-rare foci of demyelination; 2-a few foci of demyelination; and 3-large (confluent) areas of demyelination. All slides were coded and read in a blinded manner.

### Immunohistochemical analysis

Serial sections from eye, optic nerve, and brain were stained by the avidin-biotin-immunoperoxidase technique (Vector Laboratories) using 3, 3- diaminobenzidine as substrate, and a 1:100 dilution of anti-Iba1 (Wako, Richmond, VA, USA), and 1:40 dilution of antiviral nucleocapsid antiserum (mouse monoclonal anti-N; Nucleocapsid protein of MHV-JHM, monoclonal clone 1-16-1, Kindly provided by Julian Lebowitz, Texas A&M, College Station, TX) as primary antibodies. Control slides from mock-infected mice were incubated in parallel.

### Quantification of RGC numbers

RGC immunolabeling and counting was performed as in prior studies (Khan et al., 2017). Briefly, eyes removed at the time of sacrifice were fixed with 4% PFA. Retinas were isolated and whole-mounted on glass slides, washed several times in PBS, permeabilized at −70°C in 0.5% Triton X 100 solution, then thawed and washed again in 0.5% Triton X 100. Retinas were incubated overnight with goat anti-Brn3a (RGC marker) antibody (Santa Cruz Biotechnology) diluted 1:100 in blocking buffer (PBS containing 2% bovine serum albumin and 2% Triton X 100). After washing in PBS, retinas were incubated for 1 h with alexa fluor-488 anti-goat secondary antibody diluted 1:500. Retinas were then washed and mounted with vectashield mounting medium for fluorescence. Photographs of RGCs were taken in 12 standardized fields at 1/6, 3/6, and 5/6 of the retinal radius from the center of the retina in four quadrants at 40X magnification. RGCs were counted in each field by a blinded investigator using Image-Pro Plus 5.0 (Media Cybernetics, Silver Spring, MD) software.

## Results

### Intracranial, but not intranasal, inoculation with RSA59 induces spinal cord demyelination

**Figure 1:**
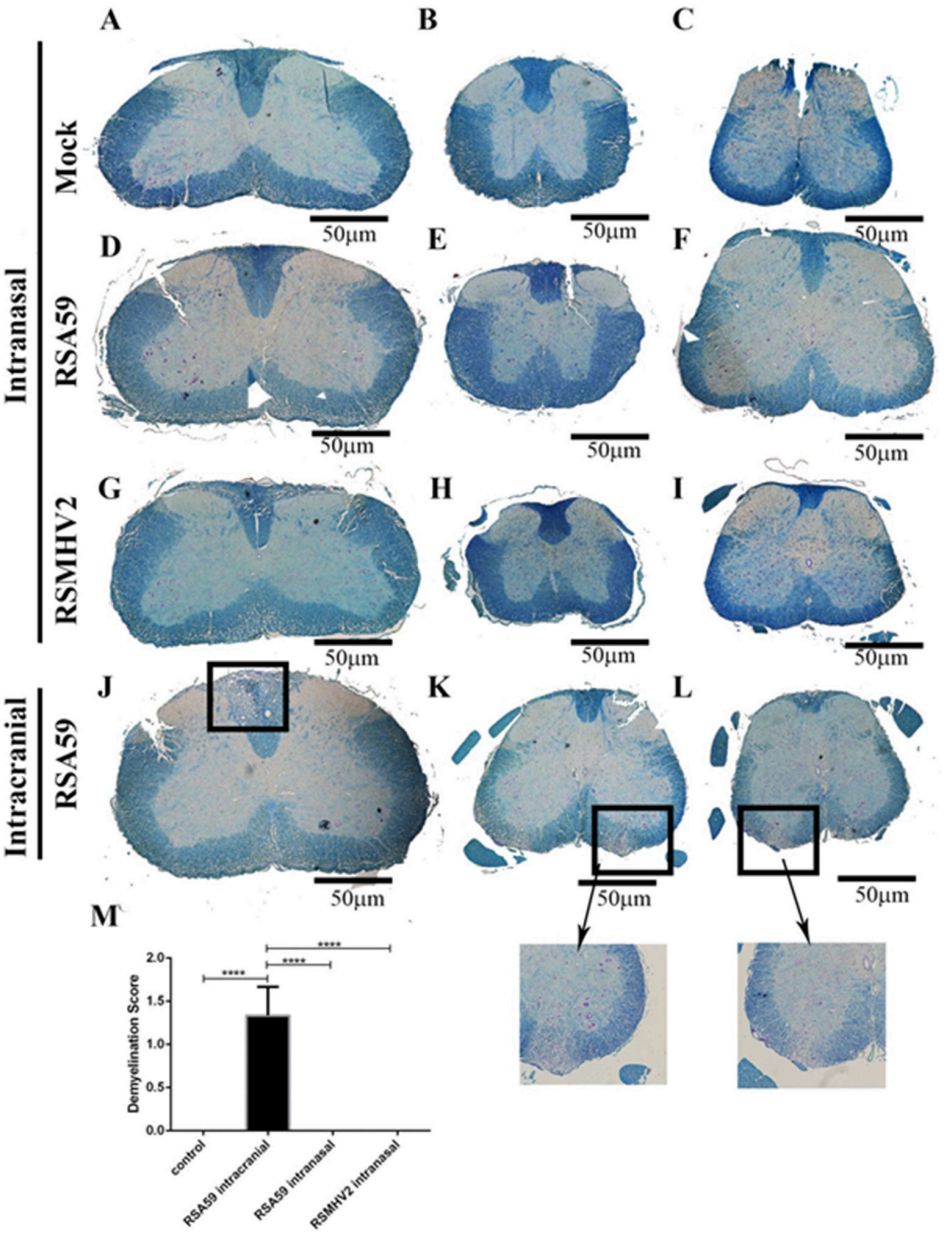
Comparative demyelination study between intracranially inoculated and intranasally infected mouse spinal cords. Serial thoracic (left column), cervical and lumbar (center and right columns) cross sections of spinal cord from post-inoculation day 26 intranasally mock-infected **(A–C)**, RSA59-infected **(D–F)**, and RSMHV2-infected **(G–I)** mice, and intracranially RSA59-inoculated mice **(J–L)** were stained with LFB (scale bar = 50 μm). Marked area indicate typical demyelinating plaques found in spinal cord white matter. Average demyelination score is 1.33 ± 0.2357; (mean ± SE) **(M)**; *n* = 9/group; data comparisons were done by one-way ANOVA and Tukey's Multiple Comparison *post-hoc* testing with GraphPad Prism 6.0 software. ****p < 0.0001.

Four week old C57BL6/J mice were inoculated with 50% LD50 doses of RSA59 or RSMHV2 by intranasal administration or by intracranial injection, or mock transfected by intranasal administration of solution without virus. Pathology was assessed from LFB stained cross-sections of spinal cord isolated from mice at day 26 (peak of demyelination) p.i. RSA59, when injected intracranially, induced significant myelin loss within formed demyelinating plaques, [average demyelination score 1.33 ± 0.2357; (mean ± SE); *n* = 3 mice (9 sections/group); *p* < 0.0001 vs. control] as in prior studies (Das Sarma et al., 2008) (Figures 1J–L). As expected, mice infected intracranially with RSMHV2 did not show any significant myelin loss (data not shown). Interestingly, no demyelination plaques were observed in any level of spinal cord sections of RSA59 infected mice when given via the intranasal route (Figures 1D–F). Similarly, as expected, no myelin loss was observed in intranasally mock-infected (Figures 1A–C) or RSMHV2-infected mouse spinal cord (Figures 1 G–I).

### Intracranial, but not intranasal, inoculation with RSA59 induces optic neuritis

**Figure 2:**
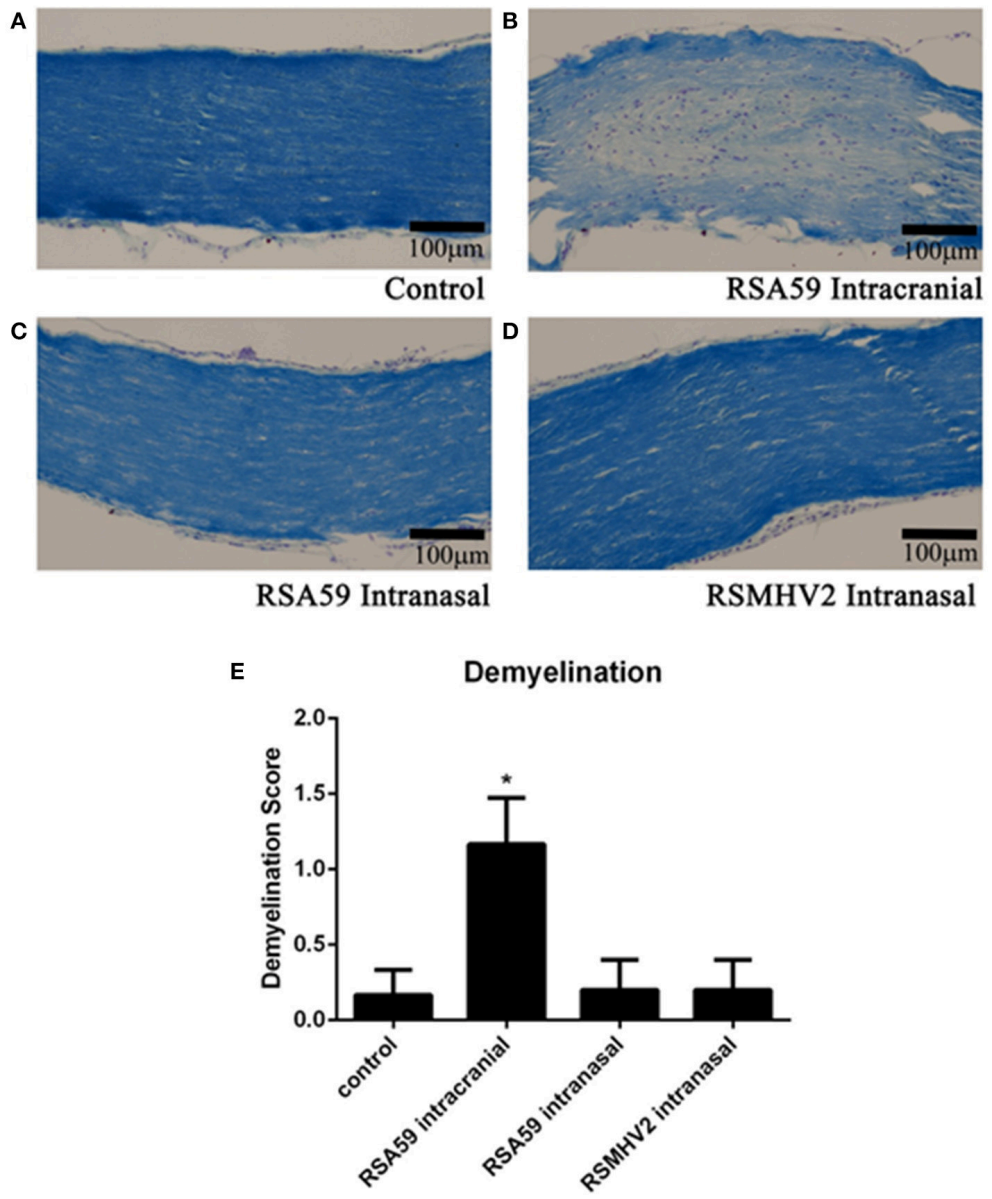
Comparative optic nerve demyelination study between intracranially-and intranasally-infected mice. Representative optic nerve sections from chronic stage (day 26 p.i.) mock-infected (*n* = 6) **(A)**, RSA59 intrancranially-infected (*n* = 6) **(B)**, RSA59 intranasally-infected (*n* = 5) **(C)**, and RSMHV2 intranasally-infected (*n* = 5) **(D)** mice stained with LFB show demyelination only in RSA59 intracranially-infected mice (scale bar = 100 μm). The relative level of demyelination scored by a blinded investigator showed significant demyelination in optic nerves of mice inoculated intracranially with RSA59, but not in mock-infected (control) mice nor in mice infected intranasally with either RSA59 or RSMHV2 (*p < 0.05 vs. all other groups) **(E)**. Data comparisons were done by one-way ANOVA and Tukey's Multiple Comparison *post-hoc* testing with GraphPad Prism 6.0 software.

Mice inoculated intracranially with RSA59 have been found to exhibit retrograde axonal transport of virus from the lateral geniculate nuclei along the optic nerve into the retina, and can cause optic nerve inflammation and demyelination (Shindler et al., 2011), whereas RSMHV2 does not. To investigate whether intranasal RSA59 administration can induce optic nerve demyelination similar to intracranial inoculation, 5 μm thick serial optic nerve sections from the same mice shown in Figure 1 were stained with LFB. RSA59, when infected intracranially, induced significant myelin loss with notable demyelinating plaques in optic nerves, (Figure 2) as previously observed (Shindler et al., 2008). Similar to spinal cord, little or no demyelinating plaques were observed in optic nerve sections of RSA59 infected mice when injected intranasally, which was comparable to RSMHV2 and mock infected mice.

### RSA59 induces RGC loss

**Figure 3:**
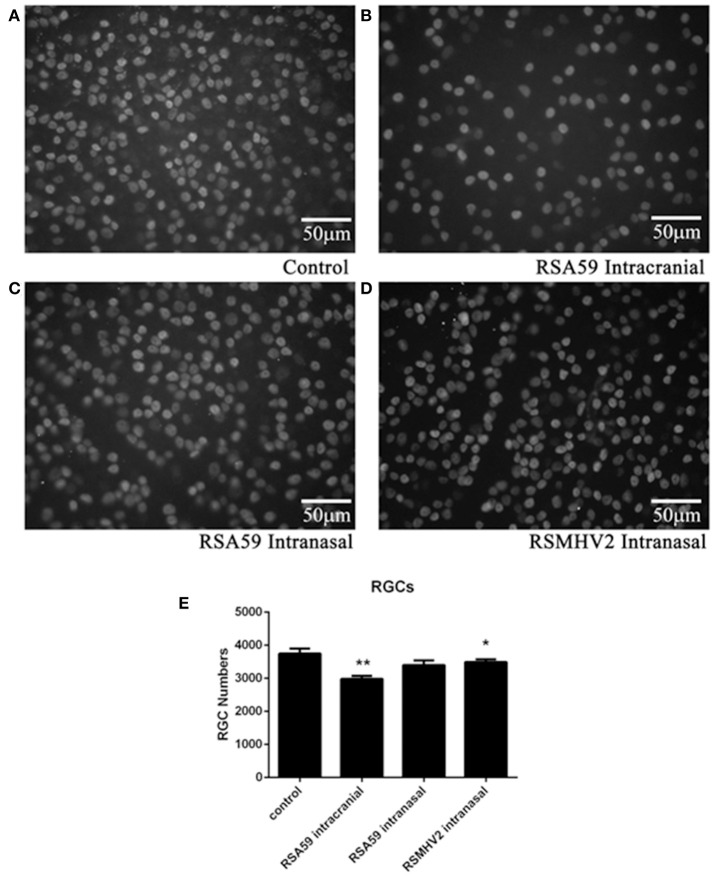
RSA59 infection induces RGC loss. Representative photos illustrate the decreased RGC numbers in eyes of mice inoculated intracranially with RSA59 **(B)** compared to mock-infected control mice **(A)**. Mice inoculated intranasally with RSA59 **(C)** or RSMHV2 did not show RGC loss **(D)** (scale bar = 50 μm). The total number of labeled RGCs present in 12 standardized retinal fields was counted. The average number of surviving RGCs/eye (*n* = 6/group) shows intracranial RSA59 induced a significant decrease in RGC numbers compared to control mice (**p < 0.01). Neither RSA59 nor RSMHV2 induced RGC loss compared to control mice when administered intranasally. RGC numbers in RSMHV2-infected mice were significantly higher than in mice intracranially inoculated with RSA59 (*p < 0.05) **(E)**. Data comparisons were done by one-way ANOVA and Tukey's Multiple Comparison *post-hoc* testing with GraphPad Prism 6.0 software.

Demyelinating optic neuritis induced by intracranial inoculation with RSA59 has been shown previously to lead to neuronal damage with loss of RGCs (Khan et al., 2014). To examine whether intranasal infection with demyelinating strains of MHV results in neuronal loss, retinas were isolated from the same mice shown in Figure 1, and RGCs were labeled and counted in a blinded fashion. Intracranially RSA59-infected mice had significantly fewer surviving RGCs compared to mock-infected mice, whereas mice infected intranasally with either RSA59 or RSMHV2 did not show RGC loss (Figure 3).

### RSA59 fails to accumulate in retina, or optic nerve acutely after intranasal inoculation

**Figure 4:**
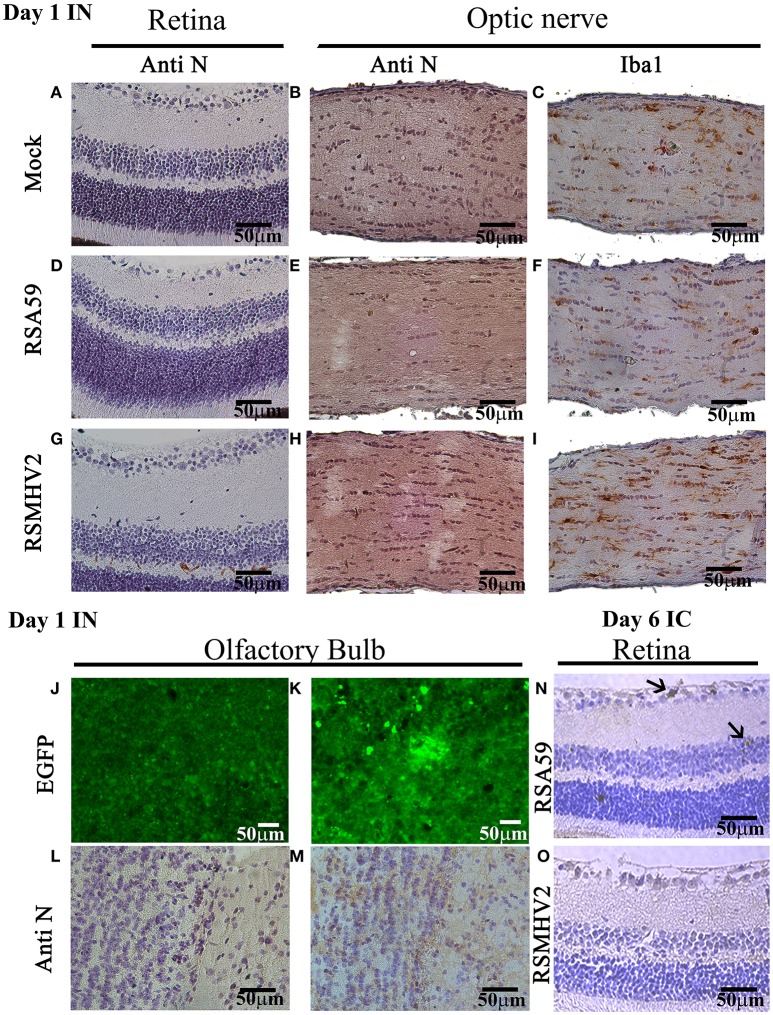
RSA59 fails to accumulate acutely in retina and optic nerve. Representative 5 μm thick sections of retina (upper left column) optic nerve (upper center and right columns) and olfactory bulb (lower panel) 1 day p.i. are shown from mock-infected mice **(A–C,J,L**) and mice intranasally-infected with RSA59 **(D–F,K,M)** and RSMHV2 **(G–I)** (scale bar = 50 μm). Retina sections 6 days p.i. from intracranially-infected RSA59 **(N)** and RSMHV2 **(O)** mice are also shown. Sections immunostained using anti-viral nucleocapsid antisera show no detectable viral antigen in retina or optic nerve on day 1 p.i. **(A,B,D,E,G,H)**, with some patchy brown stain in the olfactory bulb after intranasal viral inoculation with RSA59 **(M)**. EGFP fluorescence detected for virus presence in olfactory bulb sections shows high background in mock-infection mice **(J)** which prevented this from being quantified, but demonstrates some positive cells and clusters of cells in an RSA59 infected mouse **(K)**. Optic nerve sections stained with anti-Iba-1 antibody show the resting number and morphology of Iba-1 positive cells in mock-infected mice **(C)**, and similar resting microglial morphology across sections from RSA59 **(F)** and RSMHV2 **(I)** infected mice. Six days p.i., anti-viral nucleocapsid positive cells are present in the RGC layer (top arrow) and inner nuclear layer (bottom arrow) of the retina from a mouse intracranially-inoculated with RSA59 **(N)**, but not mice inoculated with RSMHV2 **(O)**.

Proteins rapidly accumulate at high concentrations in optic nerve and in the eye following intranasal administration (Khan et al., 2017). Thus, the potential for MHV viruses to similarly spread to optic nerve and retina was assessed 1 day following intranasal inoculation. Four-week-old, MHV-free, C57BL/6J mice were inoculated intranasally with 50% of the LD50 dose of RSA59 or RSMHV2, and mice were euthanized 1 day later. Retinas and optic nerves were isolated, sectioned, and immunostained with anti-viral nucleocapsid antisera to detect viral spread. No significant staining was observed in any of the retinas or optic nerves from mock-infected (*n* = 6), RSA59-infected (*n* = 6), or RSMHV2-infected (*n* = 6) mice (Figure 4). As shown in prior studies (Shindler et al., 2011), viral antigen does not reach the retina within 1 day following intracranial inoculation with RSA59 (data not shown). Viral antigen is found in the retina 6 days after intrancranial inoculation (Figure 4), while no antigen is detectable in retina following intranasal inoculation at day 1 (Figure 4) or any later time points (data not shown). To further confirm that intranasal RSA59 administration fails to induce optic neuritis, acutely, optic nerve sections were immunostained for the microglial/macrophage marker Iba1. Previously, it has been observed that intracranial inoculation with RSA59 induces acute optic nerve inflammation containing almost entirely activated microglia/macrophages (Shindler et al., 2011) 3–6 days post-inoculation. To study whether intranasal inoculation rapidly induces similar optic nerve inflammation, optic nerve sections were stained with anti-Iba1 antibody. Sections from mock-infected mice were used to demonstrate resting levels of microglial staining. Iba-1 staining did not reveal any increased numbers of microglia/macrophages in optic nerve sections following intranasal viral inoculation as compared to mock-infected (Figure 4). To confirm that lack of viral antigen detection in the optic nerve represents a failure of the virus to spread to optic nerve and retina, and not a failure to detect viral antigen, viral antigen was also examined in olfactory bulb sections from the same mice by autofluoresence of EGFP (Figures 4 J,K) as well as immunostaining with anti-viral nucleocapsid antisera (Figures 4 L,M).

## Discussion

The current studies compared effects of RSA59 infection via two different routes of inoculation on the development of demyelinating disease in the optic nerve and spinal cord. Results suggest intracranial inoculation is the best method to induce neuroinflammation. In the current study, intracranial infection of RSA59 lead to chronic stage inflammation and demyelination in both the optic nerve and spinal cord. This result is consistent with earlier studies where intracranial inoculation of RSA59 or its parental strain MHV-A59 led to demyelination and axonal loss in spinal cord (Das Sarma et al., 2008, 2009) and optic nerve (Shindler et al., 2008, 2011). Induction of optic neuritis by intracranial inoculation of RSA59 is dependent on retrograde transport of viral antigen along RGC axons that occurs over several days and results in late stage demyelination (Shindler et al., 2011) as seen again in the current study. Together, spinal cord and optic nerve pathology observed after intracranial inoculation of RSA59 in the current study confirm that the viral titer used retains its previously identified ability to induce CNS demyelinating disease.

While intrancranial inoculation was shown previously to induce optic neuritis (Shindler et al., 2008, 2011), effects of intranasal inoculation with RSA59 on optic nerve pathology were not reported. In the current study, intranasal infection by RSA59 at the same dose used for intracranial inoculation was not able to induce any neuropathogenesis, either in optic nerve or spinal cord. Earlier studies with several other strains showed that intranasal inoculation of MHV can cause CNS disease, but much higher concentrations of virus were used than in intracranial inoculation studies. MHV-JHM enters the central nervous system (CNS) via the olfactory after intranasal inoculation (Barnett and Perlman, 1993). The intranasal inoculation of JHM strain can lead to encephalitis and demyelination. The OBLV strain of MHV can infect the main olfactory bulb (Schwob et al., 2001). Intranasal inoculation of MHV-A59 can lead to hepatitis with measurable viral load in brain as well (Hemmila et al., 2004). These studies did not report optic nerve pathogenesis following intranasal inoculation although it is not known if that was examined. There are several possible reasons that we did not see retinal infection or optic nerve inflammation and demyelination lesions after intranasal inoculation. Most likely, the dose of virus may have been too low to cause the pathogenesis. This finding was not unexpected in the spinal cord, where previous studies showed higher doses were necessary. However, based on the high levels of protein accumulation in optic nerve and eye following intranasal inoculation (Khan et al., 2017), it was anticipated that RSA59 would also preferentially accumulate in the eye, but results suggest that higher doses are likely required. Alternatively, the intranasal inoculation may be less effective for inducing optic neuritis and retinal lesions than the intracerebral route because of the longer distance required for the virus to travel to the eye if it travels via axonal transport and neuron-neuron spread similar to what has been observed following intracranial inoculation (Das Sarma et al., 2009; Shindler et al., 2011).

The precise mechanisms mediating spread of a virus or a drug from the nose to various CNS regions are not fully elucidated. At least three steps are necessary following intranasal administration (1) crossing the epithelial barrier in the nasal cavity, (2) transport from nasal mucosa to site of brain entry, likely across the cribiform plate, (3) transport from the site of brain entry to other anatomical regions. Alternatively, they may be absorbed into the systemic circulation and gain secondary access to the CNS through the blood brain barrier. The speed at which proteins were reported to reach the eye and optic nerve, 30 min after intranasal administration (Khan et al., 2017), suggests hematogenous spread is very unlikely, and that intraaxonal transport is even more unlikely. It was hypothesized that perhaps some pathway of local diffusion or lymphatic pathway may allow rapid protein diffusion over the relatively short distance from absorption through the cribiform plate to optic nerve (Khan et al., 2017). For RSA59, prior intracranial studies demonstrate that the virus can use axonal transport machinery to spread intraneuronally (Das Sarma et al., 2009), thus it is likely that a similar mechanism would be used after intranasal administration and entry into the olfactory nerve. The path from there to optic nerve is not direct, and thus may require much higher titers of virus to occur at a pathologic level, or may not be possible at all. These hypothesized mechanisms may explain why we were not able to see viral staining day 1 post infection whereas the drug ST266 and other small molecules can be found in optic nerve as early as 30 min post intranasal inoculation (Dhuria et al., 2010; Khan et al., 2017).

Intranasal administration provides a potential non-invasive method for delivering material to the CNS. Interestingly, complex mixtures containing physiologic concentrations of multiple proteins show that protein can rapidly accumulate in the eye and optic nerve following intranasal delivery, suggesting a direct nose to eye diffusion pathway that remains to be fully elucidated (Khan et al., 2017). The current results show that RSA59 does not follow a similar pattern of accumulation in the optic nerve, suggesting that viral particle may be too large or complex to follow the same pathway, or may actively enter neurons locally and restrict their movement to intraneuronal axonal transport. Nonetheless, the ability of neurotropic MHV viruses to infect different cells, translocate throughout the CNS, and induce inflammatory demyelination, continues to provide a reproducible model to study optic nerve and spinal cord demyelinating disease following intracranial inoculation. Thus, intracranial inoculation should continue to be considered a preferred method for studies of MHV-induced optic neuritis and CNS demyelinating disease.

## Author contributions

MS, RK and KD performed the wet lab experiments and wrote their contributions. KS and JD designed the studies and wrote the paper.

### Conflict of interest statement

The authors declare that the research was conducted in the absence of any commercial or financial relationships that could be construed as a potential conflict of interest. The reviewer SM and handling editor declared their shared affiliation at time of review.
